# Inappropriate expression of the translation elongation factor 1A disrupts genome stability and metabolism

**DOI:** 10.1242/jcs.192831

**Published:** 2016-12-15

**Authors:** Daniel J. Tarrant, Mariarita Stirpe, Michelle Rowe, Mark J. Howard, Tobias von der Haar, Campbell W. Gourlay

**Affiliations:** 1 Kent Fungal Group, School of Biosciences, University of Kent, Canterbury, Kent CT2 7NJ, UK; 2 Department of Biology and Biotechnology, Sapienza, University of Rome, 00185 Rome, Italy

**Keywords:** eEF1A, Amino acid regulation, Vacuole, Genome stability, Spindle pole body, Actin cytoskeleton, Oncogene, Trehalose

## Abstract

The translation elongation factor eEF1A is one of the most abundant proteins found within cells, and its role within protein synthesis is well documented. Levels of eEF1A are tightly controlled, with inappropriate expression linked to oncogenesis. However, the mechanisms by which increased eEF1A expression alters cell behaviour are unknown. Our analyses in yeast suggest that elevation of eEF1A levels leads to stabilisation of the spindle pole body and changes in nuclear organisation. Elevation of the eEF1A2 isoform also leads to altered nuclear morphology in cultured human cells, suggesting a conserved role in maintaining genome stability. Gene expression and metabolomic analyses reveal that the level of eEF1A is crucial for the maintenance of metabolism and amino acid levels in yeast, most likely because of its role in the control of vacuole function. Increased eEF1A2 levels trigger lysosome biogenesis in cultured human cells, also suggesting a conserved role within metabolic control mechanisms. Taken together, our data suggest that the control of eEF1A levels is important for the maintenance of a number of cell functions beyond translation and that its de-regulation might contribute to its oncogenic properties.

## Abstract (precis)

**Summary:** The translation elongation factor eEF1A is elevated in some cancers. We use yeast and human cell models to show that eEF1A elevation leads to genome instability and metabolic alterations that might affect its oncogenic properties.

## INTRODUCTION

The eukaryotic translation elongation factor 1A (eEF1A) is one of the most abundant proteins in the cell, accounting for between 3 and 10% of all soluble protein (Merrick, 1992). It is an essential translation elongation factor which, when bound to GTP delivers aminoacylated-tRNA to the A site of the ribosome. The interaction between mRNA and cognate tRNA within the A site of the ribosome activates the GTPase activity of eEF1A leading to the release of its aminoacyl tRNA to the A site of the ribosome. eEF1A-GDP is then released to be recycled back to its GTP-bound state, allowing it to participate in further rounds of elongation. Alongside this canonical role in translation, eEF1A has been reported to be involved in a number of other important cellular functions including actin bundling, nuclear export, apoptosis and the induction of tumour growth (Grosshans et al., 2000; Munshi et al., 2001; Thornton et al., 2003). However, despite its importance, the mechanisms by which eEF1A participates in roles beyond translation remain largely uncharacterised.

The yeast *Saccharomyces cerevisiae* possesses two identical eEF1A-encoding genes *TEF1* and *TEF2* (Schirmaier and Philippsen, 1984). In contrast, eEF1A exists as two variant tissue-specific isoforms, eEF1A1 and eEF1A2, in all vertebrates. The first isoform, eEF1A1 is expressed in all tissues during development but is no longer detectable in the muscles and heart tissue of adults (Lee et al., 1992; Chambers et al., 1998). Instead high-level expression of eEF1A2 is switched on in these tissues, as well as in motor neurons of the medulla (Newbery et al., 2007). The loss of eEF1A2 in mice results in the mutant ‘wasted mouse’ phenotype with symptoms including weight loss, tremors, progressive atrophy of the spleen and thymus leading to death (Chambers et al., 1998)*.* There is also strong evidence implicating eEF1A2 as a bona fide oncogene (Anand et al., 2002). Its levels are elevated in a number of cancer types including breast, ovarian and lung cancers and are correlated with disease progression, decreased lifespan and a poor prognosis (Li et al., 2006; Pinke et al., 2008; Kallioniemi et al., 1994; Anand et al., 2002; Lam et al., 2006). The overexpression of eEF1A2 in Swiss NIH3T3 cells results in an enhanced growth rate, anchorage-independent growth and an induced tumour formation when xenografted in nude mice (Anand et al., 2002). Depletion of eEF1A2 in lung cancer models, using short-interfering RNA, reduces cell proliferation and promotes apoptosis (Pinke et al., 2008). Given these observations it is likely that eEF1A isoforms participate in a number of, as yet uncharacterised, cell processes that link cell growth with the process of protein translation.

One example of this is the role that eEF1A is known to play in the control of cytoskeletal stability. eEF1A has a conserved role as an actin-binding protein and this activity has been observed in the yeasts *S. cerevisiae* and *Schizosaccharomyces pombe*, the slime mould *Dictyostelium discoideum* and in mammalian cell systems (Yang et al., 1990; Edmonds et al., 1996; Suda et al., 1999; Gross and Kinzy, 2005). eEF1A not only binds but can also cross-link F-actin, and in doing so generates actin bundles that possess a unique structure excluding all other actin cross-linkers (Owen et al., 1992). Genetic manipulations of eEF1A have begun to elucidate the mechanism of the eEF1A interactions with actin, with residues in domains II and III shown to be important for the bundling activity (Gross and Kinzy, 2005, 2007). Studies have revealed two classes of eEF1A mutations that exhibit separable actin binding and translation elongation functions. The first are those which do not affect the rate of protein synthesis, but result in a disorganised actin cytoskeleton and reduced actin bundling (Gross and Kinzy, 2005). The second class of mutations disrupt actin dynamics, leading to a reduction in growth rate and a decreases in levels of translation initiation (Gross and Kinzy, 2007). The binding sites on eEF1A for aminoacylated -tRNA and actin have been shown to overlap (Liu et al., 1996), leading to the suggestion that actin binding and translation activities might be mutually exclusive and that two pools of eEF1A might exist within cells, one that is actin bound and translation incompetent, and one that is actively involved in translation. Further to its role in the regulation of the cytoskeleton, eEF1A has the ability to bind to microtubules and influence their stability both *in vitro* and *in vivo* (Shiina et al., 1994; Moore et al., 1998; Tong et al., 2005).

In this study, we re-visit the cellular consequences of eEF1A elevation in both yeast and human cells. We find that the elevation of eEF1A levels affects growth and nuclear organisation in both systems. Our data obtained in yeast suggest a new and uncharacterised role for eEF1A in the stability of the spindle pole body and a strong synthetic interaction with the dynactin complex. In addition, our analyses of global gene expression profiles reveal that the elevation of eEF1A levels leads to metabolic changes that are indicative of cell stress. In addition, vacuolar defects associated with increased eEF1A levels are associated with a loss in the regulation of amino acid homeostasis. These effects occur independently of translation, which does not appear to be affected by elevation of eEF1A levels. Our data therefore reveal new insights into the cellular changes that accompany the loss of regulation in eEF1A levels and suggest new mechanisms that might be relevant to the oncogenic properties of this protein.

## RESULTS

### Increased eEF1A levels disrupt nuclear organisation and promote senescence

**Fig. 1.**
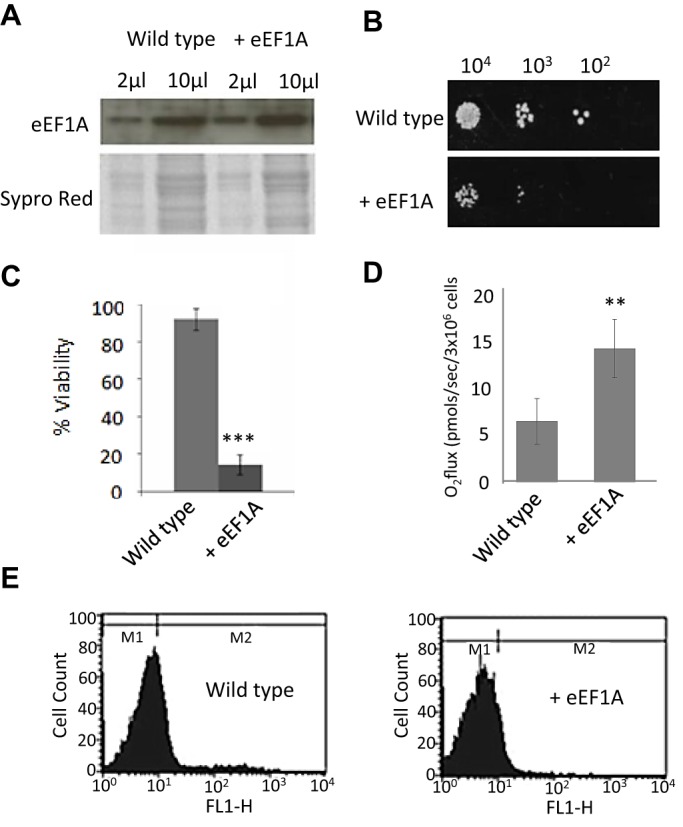
**Overexpression of eEF1A in yeast cells promotes senescence.** (A) An empty multi-copy control plasmid or one containing eEF1A under the control of a GPD promoter was introduced into a wild-type yeast strain and grown on selective medium. Levels of eEF1A were assessed by western blotting following protein extraction using a quantitative method (von der Haar, 2007). Two different volumes were loaded to help assess changes in eEF1A level, which were measured by densitometry using ImageJ with loading differences corrected using Sypro Red staining. (B) The effects of eEF1A overexpression were assayed by spotting a serial dilution series of cells taken from cultures grown to log phase. (C) Viability of cells within a culture grown for 24 h to early stationary phase in synthetic medium lacking leucine+2% glucose was assessed by a colony-forming unit assay, *n*=3. (D,E) Oxygen consumption was measured using a high-resolution respirometer (D) and ROS production was measured by flow cytometry using H_2_DCF-DA (E) after 24 h growth to early stationary phase in synthetic medium lacking leucine+2% glucose (*n*=3). Error bars represent standard deviation. **P<0.01, ***P<0.001 (Student's *t*-test).

Given the increasing number of roles assigned to eEF1A beyond translation, we sought to further characterise the effects of its overexpression. To achieve this we introduced additional copies of the eEF1A-encoding gene *TEF1* on a plasmid into wild-type yeast cells. Our construct led to a reproducible and stable increase in eEF1A protein level of ∼80% (*n*=3, Fig. 1A). This is a relatively small increase given that the 2 µ plasmid employed for overexpression has a typical copy number of 50–100 copies per cell. Quantitative real-time PCR (qPCR) assays showed that plasmids containing the *TEF1* gene had a strongly reduced copy number compared to the empty control plasmid (data not shown), consistent with strong selective pressure against overexpression of this gene (Moriya et al., 2006). As expected, the overexpression of eEF1A resulted in fewer and slower growing colonies when compared to control cells (Fig. 1B). In liquid growth medium, eEF1A overexpression led to both a reduction in growth rate and an increase in the lag phase prior to growth (Fig. S1A,B). In agreement with published data, the ribosomal profiles of actively growing cells overexpressing eEF1A were indistinguishable from controls suggesting that reduced growth was not a result of gross changes in the process of translation (Fig. S2). Interestingly, eEF1A overexpression led to a significant reduction in viability as cells entered the stationary phase of growth (Fig. 1C). This loss of viability was accompanied by an increase in respiration (Fig. 1D) and a lack of cell death markers, such as the accumulation of reactive oxygen species (ROS) (Fig. 1E). This suggests that increased levels of eEF1A promote a senescent state as cells experience nutritional depletion.

### eEF1A influences dynactin complex stability and spindle assembly

**Fig. 2.**
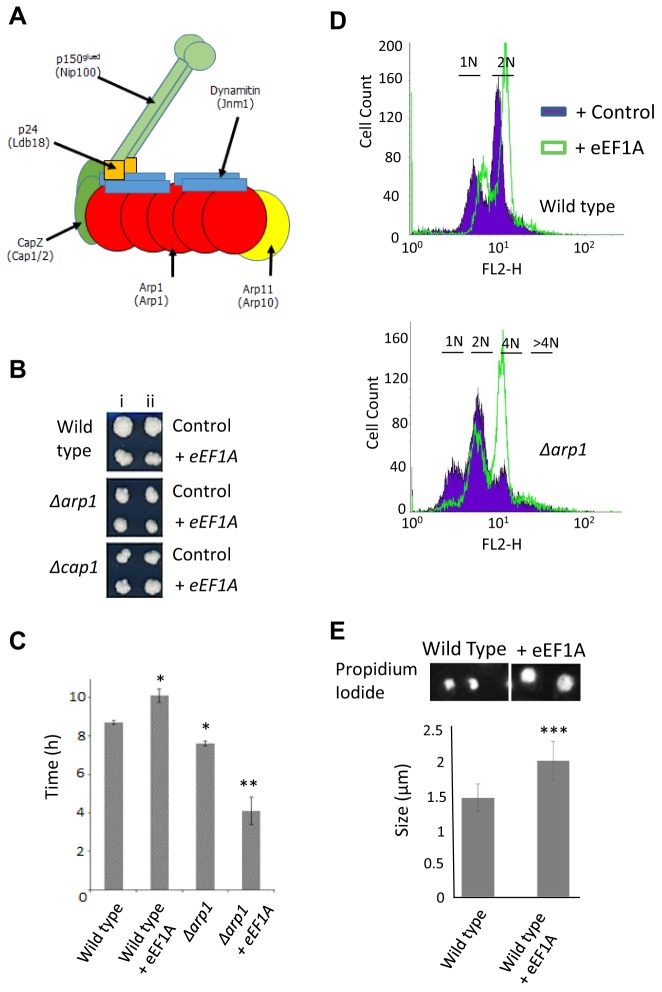
**Increased eEF1A levels promote genome instability in yeast.** (A) A schematic of the yeast dynactin complex is presented. (B) The effects of eEF1A overexpression upon colony size are shown in wild-type, *Δarp1* and *Δcap1* cells; two independent transformants are shown (i and ii). (C) The effect of eEF1A overexpression on time taken to initiate growth following inoculation, also known as the lag phase, were calculated in wild-type and *Δarp1* cells (*n*=3, mean±s.d.). (D) DNA content was analysed in wild-type or *Δarp1* cells containing a control (blue filled) or eEF1A overexpression (green open) plasmid using flow cytometry following propidium iodide staining. (E) Propidium-iodide-stained nuclei from wild-type or *Δarp1* cells containing a control or eEF1A overexpression plasmid were visualised using fluorescence microscopy and their size determined using ImageJ software (*n*=100 mean±s.d.). *P<0.05, **P<0.01, ***P<0.001 (Student's *t*-test).

eEF1A is known to interact with the cytoskeleton and has a well-defined ability to bind and bundle actin filaments (Gross and Kinzy, 2005; Owen et al., 1992; Yang et al., 1990). To address whether elevated levels of eEF1A exhibit a synthetic interaction with processes linked to actin function, we overexpressed it in a panel of yeast strains deleted for non-essential genes associated with actin organisation or actin-related processes (data not shown). Within this screen, we observed synthetic interaction with strains lacking components of the dynactin complex, which has an essential dynein-activating activity and facilitates transport of microtubular-associated cargo. The dynactin complex contains the actin-related protein 1 (Arp1), which forms an octameric polymer that terminates at its ‘barbed’ end with the actin-capping protein CapZ (a complex of Cap1 and Cap2 in yeast) (Schafer et al., 1994) with a further actin-related protein, Arp11, placed at the opposite end (Eckley et al., 1999). Projecting from the Arp1 rod is the flexible and extendable side arm that is made up of the remaining three subunits, Nip100 (p150glued or DCTN1 in mammals), Jnm1 (dynamitin in mammals) and Ldb18 (p24, also known as dynactin 3 in mammals) (Fig. 2A). As an example of this synthetic interaction, we show that overexpression of eEF1A did not result in a further reduction in colony size, as is observed in wild type, in cells lacking the dynactin complex components *ARP1* or *CAP1* when compared to controls (Fig. 2B). In addition, the deletion of *ARP1* led to a reduction in time taken to initiate growth (lag phase), which was further reduced upon eEF1A overexpression (Fig. 2C).

Dynactin is known to participate in chromosome alignment and nuclear positioning and it has been shown that the deletion of dynactin subunits results in aberrant spindle pole body positioning and chromosome segregation (Maruyama et al., 2002; Yeh et al., 2012). We therefore assessed whether changes in eEF1A levels led to changes in DNA content in actively dividing cells. Our results suggest that the elevation of eEF1A alone does not result in an increase in aneuploidy (Fig. 2D; Table S2). However, it was observed that all peaks in the fluorescence-activated cell sorting (FACS) spectrum shifted substantially upon eEF1A elevation indicating an increase in dye uptake that might indicate altered nuclear organisation (Fig. 2D). In contrast, deletion of components of the dynactin complex did lead to an increase in aneuploidy with the emergence of a substantial 4N peak (Fig. 2D; Table S2). Interestingly, the overexpression of eEF1A-in cells lacking dynactin complex components led to a significant additional accumulation of aneuploid cells (Fig. 2D; Table S3, Fig. S3). Our data suggest that elevated eEF1A levels have a substantial translation-independent effect upon nuclear organisation. This hypothesis was confirmed by our observation that nuclei of eEF1A-overexpressing cells were considerably larger than those of wild-type control cells (Fig. 2E). These larger nuclei were observed to take up more propidium iodide stain, providing an explanation for the increase in fluorescence signal observed by FACS in wild-type cells overexpressing eEF1A (Fig. 2D).

**Fig. 3.**
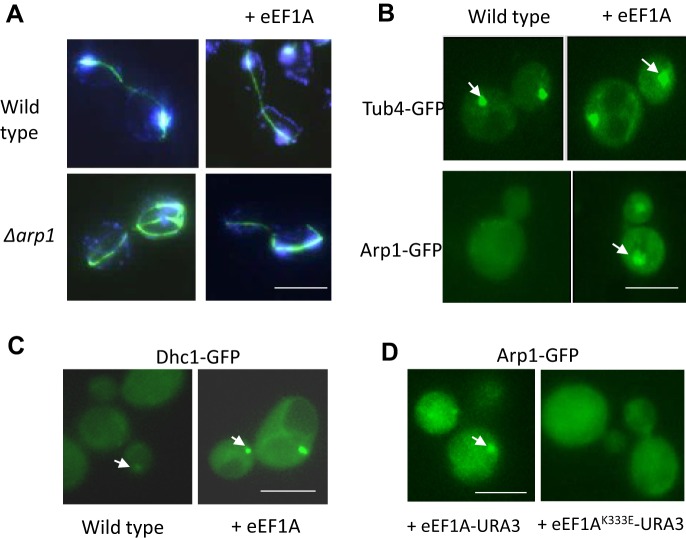
**eEF1A acts to stabilise the yeast spindle pole body.** (A) Immunofluorescence was used to visualise β-tubilin (green) and DNA (blue) in wild-type and *Δarp1* cells containing an empty or eEF1A overexpression plasmid. (B) The spindle pole body (TUB4–GFP, γ-tubulin) or ARP1–GFP (dynamin) were observed in dividing wild-type cells containing an empty control or eEF1A overexpression plasmid. (C) GFP-labelled dynein heavy chain (Dhc1) was observed in log phase cells containing an empty control or eEF1A overexpression plasmid. (D) A construct expressing eEF1A–URA3 or eEF1A^K333A^ was introduced into wild-type cells, and ARP1–GFP was visualised using fluorescence microscopy. Arrows identify proteins localised to the spindle pole body in all cases. Scale bars: 10 µm.

Next, we checked whether elevated eEF1A levels were associated with aberrant spindle organisation in dividing cells by analysing immunofluorescent β-tubulin staining (Fig. 3A). Spindles in wild-type yeast cells originated from the spindle pole body and extended from one tip in the mother cell to the polar opposite tip in the daughter (Fig. 3A). eEF1A overexpression alone did not grossly affect spindle formation, and dividing cells appeared similar to wild type (Fig. 3A). In contrast, cells lacking *ARP1* exhibited aberrant spindle organisation, with cells exhibiting multiple β-tubulin cables (Fig. 3A). Interestingly, eEF1A overexpression in a *Δarp1* mutant background appeared to prevent the formation of multiple spindles (Fig. 3A); however, this did not lead to a decrease in aneuploidy (Table S2). Spindle pole body positioning and organisation was examined by visualising γ-tubulin–GFP (Fig. 3B). An increase in eEF1A level resulted in the formation of correctly positioned but visibly larger and brighter spindle pole bodies in wild-type cells, suggesting stabilisation of γ-tubulin structures (Fig. 3B). An increase in eEF1A levels also led to a very large increase in the presence of Arp1 at the spindle pole (Fig. 3B). Considering the canonical roles of dynactin in microtubule anchoring at the spindle pole body and regulation of microtubule dynamics (Quintyne and Schroer, 2002), this presumably reflects an increase in overall spindle pole body stability. The sole known function of dynactin in yeast cells is to facilitate the motor activity of dynein (Moore et al., 2008; Muhua et al., 1994; Eshel et al., 1993). In support of the elevation of eEF1A stabilising the spindle pole body, we also observed an accumulation of GFP-labelled dynein heavy chain (Dhc1, also known as Dyn1) (Fig. 3C). The increased spindle pole body stability, shown here by the accumulation of Arp1 at the spindle pole body (Fig. 3D), was not observed upon expression of a mutant isoform of eEF1A, eEF1A^K333E^, that is well documented to be unable to bind to actin (Fig. 3D). Taken together, these data suggest that an excess of eEF1A leads to the stabilisation of the spindle pole body through its interaction with actin in yeast cells, which in turn affects genome integrity.

### Elevated levels of human eEF1A2 affect growth and nuclear organisation in HEK293 cells

**Fig. 4.**
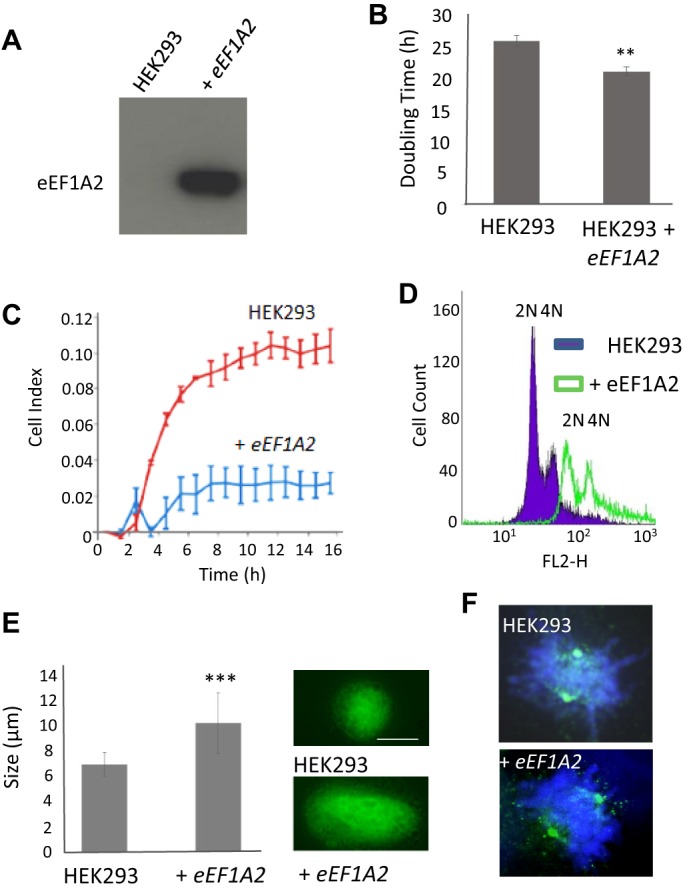
**Expression of eEF1A2 in HEK293 cells affects growth and genome stability.** (A) A stable HEK293 eEF1A2 expression cell line was generated and confirmed by western blotting. (B) The effects of eEF1A2 expression upon growth rate of HEK293 cells was assessed in biological triplicates using an xCelligence analyser, *n*=3. (C) The rate of attachment of HEK293 cells and HEK293 cells expressing eEF1A2 (HEK293+eEF1A2) to the growth surface was assessed using an xCelligence analyser. (D,E) DNA content of actively growing HEK293 and HEK293+eEF1A2 cells was assessed in fixed cells by propidium iodide staining and FACS analysis (D) and visually by fluorescence microscopy (E); nuclear size was calculated using ImageJ software (*n*=80). Scale bar: 8 µm. (F) γ-tubulin (green) distribution within mitotic HEK293 and HEK293+eEF1A2 cells were assessed by immunofluorescence; cells were co-stained with DAPI (blue). Error bars represent standard deviation. **P<0.01, ***P<0.001 (Student's *t*-test).

Elevated levels of eEF1A2 have been found in many tumours, and expression of eEF1A2 in non-native tissues is known to induce tumorigenesis (Anand et al., 2002). Given our findings in the yeast system, we generated eEF1A2-expressing human embryonic kidney 293 (HEK293) cells (Fig. 4A) to examine whether its elevation also affected the control of cell growth control and nuclear organisation. HEK293 cells were selected as they do not express eEF1A2 and so serve to highlight the effects of inappropriate expression of this isoform. Initially, we assessed the effects of eEF1A2 on growth using an Xcelligence RTCA DP Analyser. This is an automated system that allows analysis of attachment and growth of adherent cell lines by continuous measurement of the impedance of an electric current across a gold-plated well. Cell growth is measured as an increase in impedance and reported as a Cell Index (CI) value. This analysis revealed that, as has been reported to occur in NIH3T3 cells (Anand et al., 2002), an increase in eEF1A2 leads to a reproducible reduction in doubling time in HEK293 cells (Fig. 4B). HEK293 cells overexpressing eEF1A2 also initiated surface attachment more rapidly than controls (Fig. 4C) and appeared to spread less on the surface of tissue culture vessels leading to both a rounded appearance and disorganised microtubular cytoskeleton (data not shown). FACS analysis and microscopy revealed that, as had been observed in yeast, the expression of eEF1A2 did not lead to an increase in aneuploidy but did result in a substantial shift in the fluorescent signal from 2C and 4C peaks (Fig. 4D). This finding was consistent with our observation that, as had been observed in yeast, eEF1A2 cells possessed enlarged nuclei (Fig. 4E). However, in contrast to our findings in the yeast system, we did not observe differences in γ-tubulin distribution at the centrosome within mitotic HEK293 eEF1A2-expressing cells (Fig. 4F). These results suggest that the elevation of eEF1A affects both growth and nuclear architecture in HEK293 cells.

### Increased eEF1A levels modify carbon flux within yeast cells

**Fig. 5.**
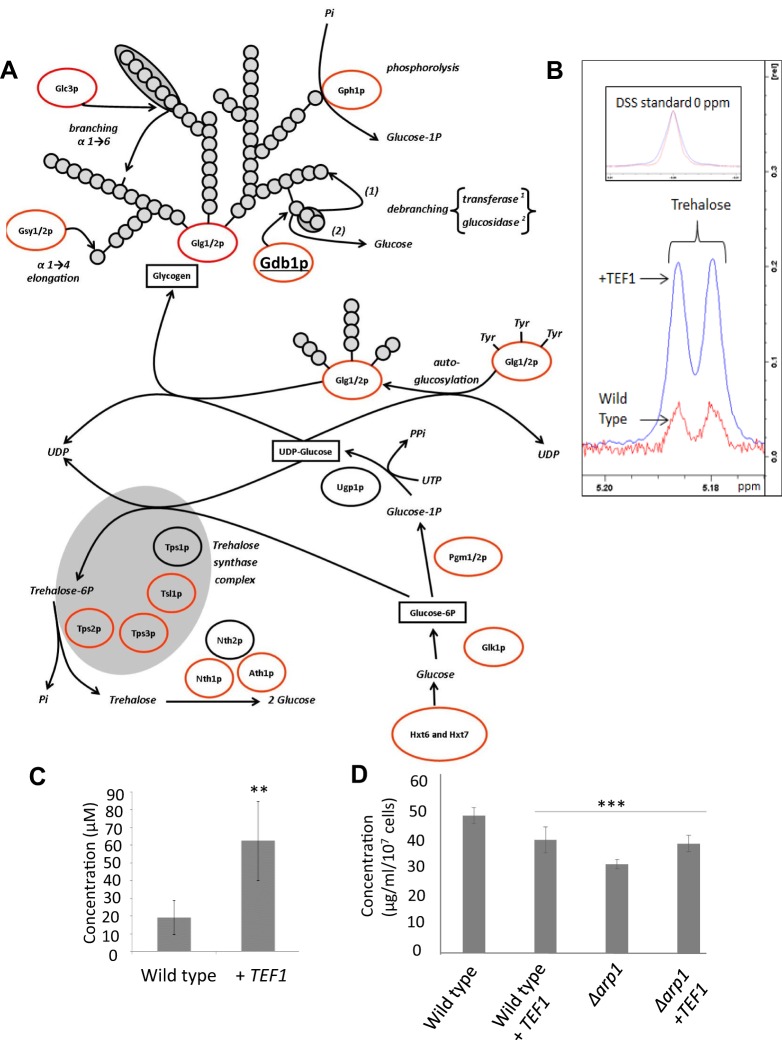
**Effects of eEF1A elevation on carbon flux in yeast.** (A) A microarray analysis was carried out in yeast cells to determine the effects of eEF1A overexpression on global gene expression during the log phase of growth. The genes identified as upregulated that are involved in the processes of glucose uptake or storage as trehalose or glycogen upon overexpression of eEF1A are circled in red. The glycogen debranching enzyme Gdb1, which possesses both α-1,4-glucanotransferase and α-1,6-glucosidase activity (Teste et al., 2000), has been underlined to highlight its upregulation. (B,C) The elevation of trehalose during log phase was confirmed using quantitative NMR; the peaks used for assignment are presented in B, and levels were quantified with reference to a known DSS standard (C). (D) The accumulation of glycogen in response to eEF1A overexpression was assessed in log phase wild-type and *Δarp1* cells using a biochemical assay as outlined in the Materials and Methods. Error bars represent standard deviation (*n*=6, C; *n*=3, D). ***P<0.001, **P<0.01 (Student's *t*-test).

Given our observations that elevated levels of eEF1A lead to genomic instability in yeast, we sought to determine its effects upon gene expression during log phase growth using a microarray approach. Upon overexpression of eEF1A we found that a total of 319 genes that were upregulated and 61 that were downregulated during log phase when a B-statistic of 1.5 was applied as a cut-off threshold (Tables S3 and S4). A number of indicators of cell stress were present within the data set including the upregulation of genes involved in the response to osmotic stress, starvation, cell wall stress and heat shock (Table S3). The most highly enriched biological processes were those of carbohydrate transport and processing (Table S3). Upon closer analysis, we found that genes involved in glucose uptake (*HXT1*, *HXT6*, *HXT7* and *HXT9*) and all steps required for its processing into the stress-linked and storage di- and poly-saccharides trehalose and glycogen were upregulated (Fig. 5A). We verified that cells overexpressing eEF1A accumulated trehalose during log phase growth using a quantitative nuclear magnetic resonance (NMR) approach (Fig. 5B,C). A threefold increase in trehalose levels in cells expressing eEF1A was observed when compared to controls (Fig. 5B,C). As a unique NMR peak could not be determined for glycogen, we analysed its levels during log phase growth using a biochemical assay (Fig. 5D). Surprisingly, despite an upregulation in glycogen biosynthesis genes, we observed that glycogen levels appeared to be lower than in wild type when eEF1A was overexpressed (Fig. 5D). This might be a result of the upregulation of the glycogen debranching and mobilisation enzymes Gdb1 and Gph1, respectively, in cells overexpressing eEF1A (Fig. 5A; Table S3). As we had observed a synthetic interaction between increased eEF1A levels and dynactin complex integrity, we also checked whether the deletion of *ARP1* induced glycogen accumulation (Fig. 5D). We observed that cells lacking ARP1 also had reduced glycogen levels when compared to wild type and that these levels were slightly raised when eEF1A was overexpressed (Fig. 5D). Taken together, these data suggest that the elevation of eEF1A in yeast during growth leads to a substantial alteration in the flow of carbon resulting in the accumulation of the stress-linked disaccharide trehalose.

### Increased eEF1A levels disrupt amino acid homeostasis and vacuole function

**Fig. 6.**
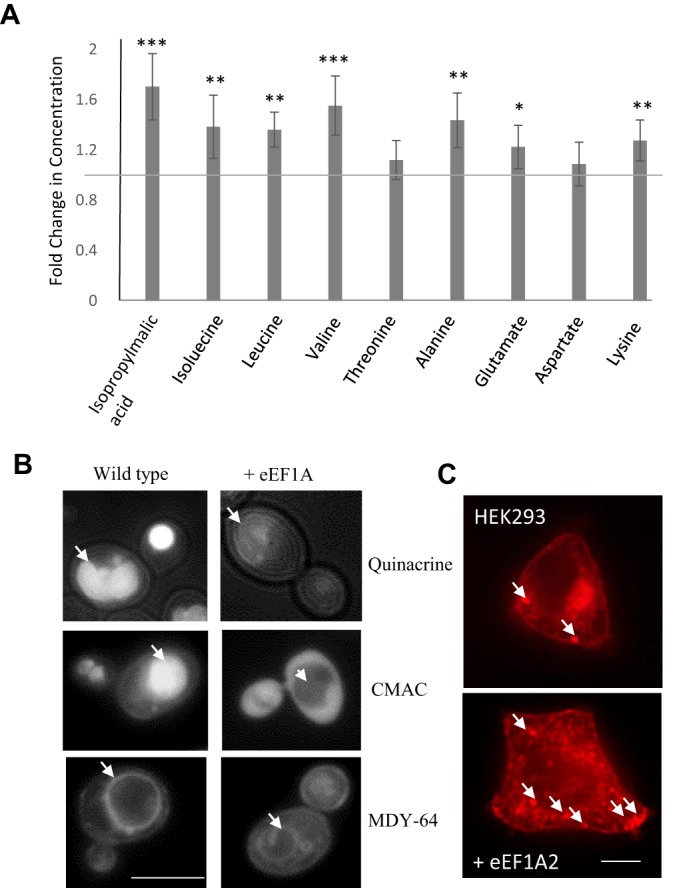
**Central metabolic control mechanisms are disrupted by eEF1A overexpression.** (A) NMR was used to quantify levels of a number of metabolites in extracts prepared from log phase wild-type yeast cells containing a control or eEF1A overexpression plasmid; data is presented as fold change compared to wild type, *n*=6. (B) Vacuole function was assessed in wild-type control and eEF1A-overexpressing cells using the fluorescent dyes Quinacrine, CMAC and MDY-64; arrows indicate the yeast vacuole. (C) Lysosomes were stained in control HEK293 or eEF1A2-expressing cells using lysosensor dye and visualised by fluorescence microscopy; arrows indicate lysosomes. Scale bars: 10 µm. Error bars represent standard deviation. *P<0.05, **P<0.01, ***P<0.001 (Student's *t*-test).

Our microarray analysis revealed an increase in a number of genes involved in the regulation of amino acid transport and metabolism (Table S3). We hypothesised that elevated levels of eEF1A might therefore impact upon the regulation of amino acid levels. To investigate this further, we employed an NMR metabolomics approach. We produced metabolite extracts from six independent actively growing yeast cultures to determine the effects of eEF1A overexpression (Fig. 6A, Table 1; Fig. S4). From the NMR spectrum, we were able to determine that the levels of a number of amino acids including isoleucine, leucine, alanine, valine, glutamate and lysine were significantly elevated upon eEF1A overexpression (Fig. 6A, Table 1). The presence of amino acid stress in cells overexpressing eEF1A is further supported within our gene expression data, as we observed the upregulation of several transporter-encoding genes, such as the aspartate transporter *AVT6* and the high-affinity branched amino acid permease *BAP2* (Table S3). Further evidence that changes in metabolic homeostasis occured upon eEF1A overexpression was that both NADH and acetic acid levels were significantly reduced (Table 1). The vacuole is the major amino acid storage and distribution organelle in yeast cells and it serves a similar purpose with respect to carbohydrates such as trehalose (Jules et al., 2004; Wilson et al., 2002). As the actin cytoskeleton plays an important role in controlling vacuole biogenesis and function (Bodman et al., 2015) and given the known role for eEF1A in controlling actin dynamics, we hypothesised that the function of this organelle might be impaired upon eEF1A overexpression. To investigate this, we made use of the fluorescent dyes MDY-64, CMAC and Quinacrine, which stain the membrane or accumulate in the lumen of functional and acidified vacuoles. In all cases the overexpression of eEF1A led to a failure to incorporate each dye to the extent observed in wild-type cells indicating vacuole dysfunction (Fig. 6B). Interestingly, a substantial increase in lysosome biogenesis was also observed in HEK293 cells overexpressing eEF1A2 (Fig. 6C). As lysosomes are the functional equivalent of the yeast vacuole this finding suggests a conserved role for eEF1A in the control of metabolism. Taken together, these data suggest that eEF1A overexpression might perturb metabolic homeostasis through interactions with the vacuolar compartment in yeast and lysosomes in human cells.

**Table 1.**
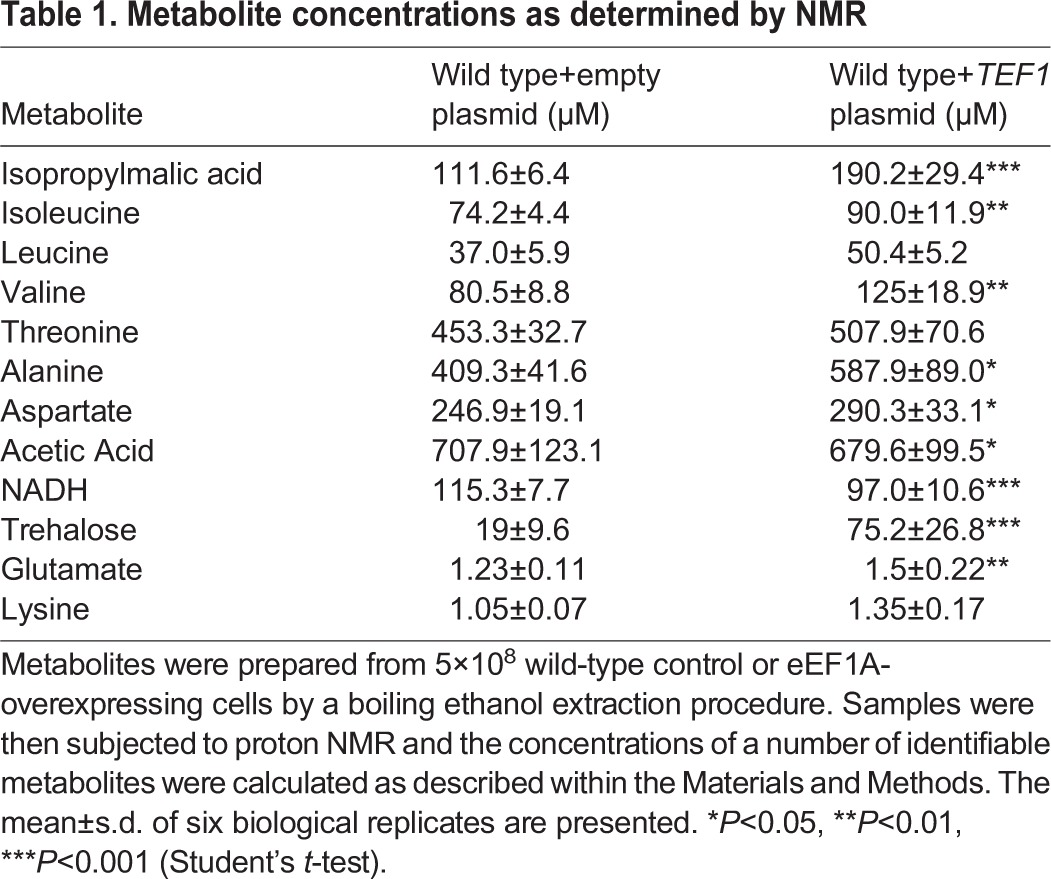
**Metabolite concentrations as determined by NMR**

## DISCUSSION

Although its canonical role in translation elongation is well characterised, the moonlighting functions of eEF1A and the effects of loss of its regulation are poorly understood. The importance of maintaining the control of eEF1A levels is highlighted by the oncogenic properties of eEF1A2, whose inappropriate expression has been shown to induce tumour growth. To increase our understanding of the consequences of an increase in eEF1A above normal levels we made use of the highly amenable budding yeast *S. cerevisiae*. Previous studies on eEF1A overexpression in yeast revealed links to altered actin cytoskeletal organisation that correlated with changes in growth (Munshi et al., 2001). These effects were attributed to the ability of eEF1A to bind to and bundle actin fibres (Gross and Kinzy, 2005), although the mechanisms that underlie its effects on growth were not identified. Here, we report that the elevation of eEF1A promotes genome instability and metabolic changes in both yeast and human cells that might contribute to its effects upon growth.

In the yeast system, we observed a strong synthetic genetic interaction between eEF1A and the dynactin complex. In higher eukaryotic systems dynactin can be found in a variety of subcellular localisations, for example, within centrosomes (spindle pole bodies in yeast) and within a variety of endomembranes (Chen et al., 2015; Gill et al., 1991; Paschal et al., 1993; Habermann et al., 2001). Dynactin is implicated in the regulation of dynein targeting and/or recruitment and facilitates its processivity along microtubules. At the centrosome, dynactin and dynein are important in microtubule anchoring (Quintyne et al., 1999), and this function is conserved in the yeast system (Muhua et al., 1994). Dynactin is also able to control microtubule dynamics and recruit proteins to the plus ends of microtubules (Quintyne et al., 1999; Yeh et al., 2012). The localisation of dynactin to the cell cortex allows it to influence rotational movement of the mitotic spindle and direct movement of motile cells (Skop and White, 1998; Gönczy et al., 1999; Dujardin et al., 2003). However, despite the plethora of known roles in higher eukaryotes, the only known function of dynactin in yeast cells is in the control of spindle pole body dynamics (Moore et al., 2008). Our data suggests that eEF1A might influence genome stability through spindle pole body stabilisation, and that this activity is revealed upon combination with additional defects in spindle pole body organisation. In line with this hypothesis, an increase in eEF1A levels dramatically increased the aneuploidy observed in yeast cells lacking core dynactin complex components. In addition, eEF1A overexpression in yeast led to the accumulation of γ-tubulin and the dynactin–dynein complex at the spindle pole body. These data indicate that an increase in eEF1A levels leads to stabilisation of the spindle pole body, with downstream effects upon nuclear organisation and genome stability. One possibility is that eEF1A is an unrecognised component of the spindle pole body, perhaps mediated by its known role as a cytoskeletal-binding protein. One can envisage that the stabilisation of the spindle pole body upon overexpression of eEF1A could arise from a stabilising effect upon actin or γ-tubulin structures therein. However, exhaustive studies on the composition of the spindle pole body in yeast have not identified it as a component part (Kilmartin, 2014; Wigge et al., 1998). It does remain a possibility that eEF1A functions within the spindle pole body under specific cellular conditions or indeed becomes incorporated when its levels are elevated.

As well as containing microtubules, the spindle pole body in yeast relies on the functions of the actin cytoskeleton for positioning. Indeed, there exists a strict cooperativity between the actin and microtubular cytoskeletons in positioning the spindle pole body to ensure faithful segregation of chromosomes. It therefore seems likely that an increase in eEF1A level perturbs nuclear stability through its role in regulating the stability of the actin cytoskeleton. In line with this, we observed that the expression of a mutant form of eEF1A, eEF1A^K333A^ which cannot bind to actin, did not lead to spindle pole body defects upon overexpression. The expression of eEF1A^K333A^ has also been shown to have no effect upon growth rate in yeast cells (Gross and Kinzy, 2005). Interestingly, the overexpression of eEF1A alone did not appear to increase the generation of aneuploid cells, as was the case upon loss of the dynactin complex, and did not affect positioning of the spindle pole body. These data suggest that the effects of eEF1A upon spindle pole body do not occur through the inactivation of dynactin function. A more likely explanation is that an actin-dependent stabilisation of the spindle pole body leads to the accumulation of dynactin and dynein with downstream effects upon nuclear organisation. However, the levels of γ-tubulin are reported to increase in response to DNA replication stress (Tkach et al., 2012). We cannot therefore rule out the possibility that the effects of eEF1A overexpression on spindle organisation and chromosomal segregation are also influenced by a stress response that in turn leads to spindle pole body stabilisation. A role for actin in the regulation of chromosome dynamics in yeast has recently been described in which both cytoplasmic and nuclear actin structures have been implicated (Spichal et al., 2016). An extension of these findings is that stabilisation of actin structures through eEF1A overexpression might alter the dynamic interplay between chromosomes and the nuclear envelope in addition to stabilisation of the spindle pole body. This relationship might in turn have an effect upon entry into the cell cycle, as we observe substantial changes in the lag phase of growth when eEF1A is overexpressed. It might be that such a role is conserved, as we also noted changes in nuclear morphology in HEK293 cells expressing eEF1A2. In both yeast and HEK293 cells, we observed that eEF1A overexpression led to an increase in nuclear size. This size increase was accompanied by an increase in propidium iodide staining, a phenomena that has been reported to occur upon a reduction in histone occupancy, which in turn reveals more dye-binding sites (Giangarè et al., 1989). It will be of interest to fully characterise the nuclear changes that occur as a result of eEF1A overexpression and to determine whether these findings are linked to the oncogenic properties of eEF1A2.

Our analyses demonstrate that increasing eEF1A levels leads to the coordinated upregulation of glucose uptake and its storage as trehalose. This raises the possibility that spindle pole body stabilisation might in turn trigger carbohydrate storage, perhaps through changes in gene expression leading from changes in chromatin. Indeed, it has been reported that DNA damage can lead to the activation of storage carbohydrate synthesis in yeast (Kitanovic et al., 2009). However, the deletion of *ARP1*, which leads to severe defects in spindle organisation and chromosome segregation, did not lead to the accumulation of glycogen when compared to wild type. Similar results have been obtained for other strains lacking dynactin complex components (data not shown) suggesting that another mechanism might be responsible for the changes in metabolic activity observed. Recent data suggests that the *TPS1* gene, which encodes the synthase subunit of trehalose-6-P synthase and which is upregulated in response to eEF1A overexpression, plays an important role in managing diverse stress responses and protection against apoptosis independently of its role in trehalose biosynthesis (Petitjean et al., 2015, 2016). This might be important given our finding that eEF1A overexpression does not lead to an increase in cell death despite its effects on nuclear stability, but instead increases metabolic functions within the cell.

Our metabolomic and gene expression profiling data suggest that the elevation of eEF1A levels leads to changes in the levels of a number of amino acids within dividing cells. The vacuole represents an important organelle that is a site for the storage of amino acids and carbohydrates. The processes of uptake and release from the vacuole are active and require the function of membrane-localised transporters (Kucharczyk and Rytka, 2001). Our data suggest that the function of the vacuole becomes impaired upon the expression of eEF1A, and our preliminary findings suggest that, under these conditions, vacuoles are found to contain amino acid levels that differ significantly from that of wild type (data not shown). Whether amino acid storage, transport or sensing mechanisms underlie the defects induced by an increase in eEF1A levels requires further investigation. A possible mechanism to explain the loss of control of vacuole function might rest with the ability of eEF1A to stabilise actin filaments. The function of the vacuole has been shown to be linked to actin filament construction, which is important for maintaining organelle stability. In support of this hypothesis, it has recently been published that eEF1A and actin interact to control vacuole stability in yeast (Bodman et al., 2015). A model is proposed whereby eEF1A binds to the small GTPase Rho1p on the vacuolar membrane. Upon activation of Rho1, eEF1A is released and acts to stabilise the vacuole through its actin-bundling activity (Bodman et al., 2015). One possibility is, therefore, that elevation of eEF1A leads to vacuolar defects through a similar mechanism resulting in an inability to control amino acid levels. However, as we do not observe vacuolar morphological defects, but instead a loss of membrane potential and acidification, it might be the case that eEF1A interacts directly or indirectly with the v-ATPase to inhibit its function. Interestingly, expression of eEF1A2 in HEK293 cells led to a dramatic accumulation of lysosomes when compared to controls. This result requires further investigation but might suggest the induction of autophagy, which results in lysosome proliferation, or alternatively the activation of a lysosome biogenesis programme. It is unclear whether an increase in eEF1A levels perturbs vacuole and lysosome functions through a similar mechanism or whether these results reflect more general responses to metabolic disruption. Interestingly, and in line with a role for eEF1A2 as an oncogene, the upregulation of lysosome biogenesis has been reported to be important for the support of cancer cell metabolic requirements (Perera et al., 2015).

Our experiments have identified two new apparently distinct changes in cell behaviour that are associated with an increase in eEF1A levels. The discovery that nuclear organisation and metabolic homeostasis are directly influenced by eEF1A levels might be of importance with respect to its ability to act as an oncogene. These effects might be attributable to the role that eEF1A plays in the stabilisation of cytoskeletal structures. It will be important to further characterise the roles and interactions of eEF1A if we are to fully understand its oncogenic properties.

## MATERIALS AND METHODS

### Strains and cell growth

*Saccharomyces cerevisiae* strains were grown at 30°C, with liquid cultures grown with rapid aeration with shaking at 180 rpm in either YP or synthetic defined medium supplemented with 2% glucose. For all experiments, cells were grown overnight in selective medium and sub-cultured to an optical density at 600 nm (OD_600_) of 0.1 before growth analysis. Yeast strains used in this study are described in Table S1. GFP-labelled yeast strains were generated as part of a genome-wide tagging project (Huh et al., 2003), and obtained from Thermo-Fisher. Growth of yeast in liquid culture was carried out in BMG labtech SPECTROstar Nano plate readers in 24-well or 48-well plates as indicated at a constant temperature of 30°C with orbital shaking at 400 rpm and automated OD_600_ determination every 30 min. To generate an eEF1A yeast expression vector, a gateway cloning LR reaction was carried out using pENTR/TEV/D-TOPO TEF1 [Havard Medical School PlasmID repository (clone ID ScCD00011628)] and pAG425GPD-ccdB. Plasmids expressing Ura3-eEF1A and Ura3-eEF1AK^333A^ were a kind gift from Stephane Gross (Aston University, Birmingham, UK). To create the eEF1A2 FlipIn HEK293 strain, a pcDNA3.0 overexpression plasmid containing eEF1A2 (a kind gift from Jonathan Lee, University of Ottawa, Canada) was digested with HindIII and XhoI, and the resultant eEF1A2 ligated into pcDNA5/FRT (Invitrogen). Stable HEK293 cell lines were generated using the Invitrogen Flp-In Recombination System, as per the manufacturer's guidelines. HEK293 cells were grown in a static incubator at 37°C and 5% CO_2_ in Dulbecco's modified Eagle's medium (DMEM), High Glucose, GlutaMAX (Invitrogen, 6195-026) with 2 mM glutamine (Invitrogen, 21765-029), 10% fetal bovine serum (FBS; Invitrogen 16000-044) and hygromycin (100 µg/ml) in T75 flasks. HEK293 cells were diluted to 5×10^4^/ml and 100 μl applied to the wells of an Xcelligence E-plate for assessment of growth in an RTCA DP analyser (ACEA Bioscience Inc.).

### Polysome profiles

For the generation of polysome profiles, cycloheximide was added at a final concentration of 0.1 mg/ml to logarithmically growing yeast cultures (OD_600_ 0.8–0.9) for 5 min prior to harvest. 60 OD_600_ units of cells were harvested by centrifugation and washed in ice-cold lysis buffer (25 mM Tris-HCl pH 7.5, 100 mM NaCl, 5 mM MgCl_2_, 5 mM β-mercaptoethanol, 0.1 mg/ml cycloheximide, 0.5 mM PMSF). Washed cells were re-suspended in 700 µl of ice-cold lysis buffer and lysed by six 60 s cycles of vortexing with a volume of acid-washed glass beads, with 60 s incubations on ice between cycles. Lysates were cleared by centrifugation at 13,000 ***g*** and 450 µl of the cleared lysate were layered onto gradients of 15–45% sucrose in lysis buffer without PMSF. Gradients were spun for 3 h at 35,000 rpm (21,000 ***g***) and 4°C, and then analysed by passaging through the OD cell of a Bandel BR-188 Gradient Fractionator (Alpha Biotech, UK).

### Whole-cell protein and metabolite extraction

Whole-cell protein extraction was performed from 10^8^ cells as described previously (von der Haar, 2007). For detection of metabolites using NMR, 50 ml yeast cultures were grown to an OD_600_ of 0.5 and a cell count was performed for quantification purposes. Cells were cooled on ice and washed twice in 25 ml of ice-cold water and the wet biomass weighed for quantification purposes. 5 ml of boiling 75% EtOH was added to the pellet together with 2 ml of 0.3 mm glass beads. The samples were vortexed for 30 s and then incubated at 80°C for 3 min followed by another 30 s vortex. Samples were decanted into a fresh tube and the beads washed with a further 2 ml of 75% EtOH that was then combined with the original sample. Samples were then centrifuged at 16,000 ***g*** for 10 min to remove cell debris and dried in a Rotorvac at 37°C. Samples were re-suspended in 330 μl of H_2_O and spun at 5000 rpm (1844 ***g***) for 10 min to remove any further debris and frozen at −20°C before being freeze dried.

### Metabolite detection by NMR

Experiments were performed at 298 K on a Bruker AVANCE 3 600 MHz spectrometer, equipped with a QCI-F cryoprobe. Data sets were acquired with 64k points and a proton window size of 16 ppm. Spectra were referenced against an internal standard of DSS. Excitation sculpting was used to suppress the water peak using pulsed field gradients. Analysis of data was performed using Bruker TopSpin and AMIX data analysis software. Identification of metabolites was performed by comparison to previously published data on the Madison Metabolomics Consortium Database and by reference to internal standards. We utilised Bruker TopSpin to quantify peak intensity with reference to a DSS standard of known concentration. Absolute concentrations were calculated using Concentration (μM)=peak X intensity/((DSS peak intensity/9 protons)×peak X protons)×50.

### Assessment of glycogen levels

Strains were grown overnight in SC-LEU medium and re-inoculated to an OD_600_ of 0.1 in fresh SC-LEU medium. Cultures were then grown to an OD_600_ of 0.5 before assessment. 10^8^ cells were washed three times in an equivalent volume of de-ionised water followed by three times in ice-cold lysis buffer (25 mM sodium citrate, 2.5 mg/ml NaF, pH 4.2) and re-suspended in 100 µl lysis buffer. An equivalent volume of acid-washed glass beads were added and cells were lysed using a vortex bead beater for three 2-min cycles before being heated to 90°C for 2 min and returned to ice. The homogenate was cleared by centrifugation at 14,000 ***g*** for 5 min and the supernatant used to assess glycogen levels with a the EnzyChrom glycogen assay kit (Bioassay Systems) as per manufacturer's instructions.

### Fluorescence microscopy

All fluorescence microscopy was performed on an Olympus IX81 inverted research microscope. Images were captured using a Hamamatsu photonics ORCA AG cooled CCD digital camera, with light excitation from an Olympus MT20 illumination system. Control of the system was through the Olympus CellR imaging software. Images were processed using Huygens deconvolution software from Scientific Volume Imaging.

### Immunofluorescence of yeast cells

Cells were grown to the desired phase of growth and fixed in 5% formaldehyde for 1 h. Cells were washed twice in sorbitol buffer (1.2 M sorbitol, 0.1 M potassium phosphate buffer pH7.5), re-suspended in 0.5 ml sorbitol buffer plus 1 μl β-mercaptoethanol and 20 μl 1 mg/ml zymolyase, and incubated at 37°C for 40 min. Cells were applied to poly-L-lysine-coated slides with wells and 10 μl of 0.1% SDS added for 30 s before washing ten times with PBS plus 1 mg/ml BSA. Immobilised cells were incubated with primary monoclonal anti-β-tubulin antibody (1:500 dilution, Sigma, clone AA2) overnight at 4°C. Slides were washed a further ten times with PBS with 1 mg/ml BSA before adding 15 μl of the secondary anti-mouse-IgG antibody conjugated to FITC (1:1000, Sigma) in PBS with BSA at room temperature for 1 h. Slides were washed ten more times and then a drop of phenylenediamine mounting solution containing DAPI at 1 mg/ml was added before visualisation.

### Immunofluorescence of HEK293 cells

HEK293 cells were grown to 70% confluence on coverslips in six-well plates before fixation with 4% paraformaldehyde in PBS for 15 min. Following fixation, cells were permeabilised with 0.1% Triton X-100 in PBS for 5 min and then blocked in 250 μl 3% BSA in PBS for 15 min at room temperature. 25 μl droplets of anti γ-tubulin (1:1000, Sigma, GTU88) primary antibody were applied to a sheet of parafilm and the coverslips were placed cell-side down on the drops and left in a moist environment overnight at 4°C. Coverslips were then placed on four 100 μl droplets of PBS with 0.1% Tween 20 sequentially and left on each for 5 min to wash. Coverslips were incubated with anti-mouse-IgG antibody conjugated to FITC (1:1000, Sigma) for 1 h at room temperature before being washed a further four times on 100 μl droplets of PBS with 0.1% Tween 20 and then placed cell-side down onto 25 μl droplets of DAPI (1 µg/ml) for 1 min followed by two 10 min washes with PBS. 100 μl 0.1% *p*-Phenylenediamine anti-fade (Sigma P6001-50G) was mixed with 900 μl 10% Mowiol mounting solution (Sigma 81381-50G), 5 μl droplets of this mounting mix were added to glass slides and the coverslips were placed, cell-side down, on top of the droplets before visualisation.

### ROS detection and propidium iodide staining

To assess ROS levels, cells were grown overnight to stationary phase in the presence of 5 mg/ml 2,7-dichloro dihydrofluorescein diacetate (H2-DCFDA; Molecular Probes). For assessment of propidium iodide staining 1×10^7^ cells were fixed in 1 ml of ice-cold 70% ethanol for 10 min before washing with 1 ml of 50 mM sodium citrate. Cells were re-suspended in 0.5 ml of 50 mM sodium citrate containing 1 mg/ml RNase A and incubated for 2 h at 37°C before the addition of propidium iodide to a final concentration of 6 µg/ml. Propidium iodide staining and ROS levels were assessed using a BD FACScalibur flow cytometer using FL-2 and FL-1 detection filters, respectively. Propidium-iodide-stained nuclei were also visualised by fluorescence microscopy using a Texas Red filter set.

### RNA isolation and microarray

Total RNA was prepared from log phase cells from biological triplicate cultures using a Qiagen RNAeasy kit including an on-column DNase digestion step according to the manufacturer's instructions. Following reverse transcription reactions, the cDNA template was hybridised to an Affymetrix Yeast 2.0 GeneChip array. Data was quality controlled and normalised using the Bioconductor plugin affylmgui (Wettenhall et al., 2006). To reduce background noise, we used the Robust Multi-Array Average (RMA) algorithm (Bolstad et al., 2003). A cut-off point was established for genes that were designated to have a B-statistic greater than 1.5. We then sorted the significant data into groups for processing with the Gene Ontology (GO) Slim Mapper. The raw and analysed microarray data have been submitted to the GEO database under accession number GSE89286.

### Western blotting procedures

Whole-cell protein extraction was performed from 108 cells as described previously (von der Haar, 2007), and samples were separated on a 12.5% SDS-PAGE gel before being transfered to a PVDF membrane at 25 V for 30 min using a semi-dry blotter (Biorad) in transfer buffer (25 mM Tris-HCl, pH 8.3, 190 mM glycine, 20% methanol). Membranes were incubated in TBST (20 mM Tris-HCl, pH 7.5, 150 mM NaCl, 0.1% Tween 20) containing 5% milk for 1 h before incubating with either an anti-human eEF1A1 antibody (1:1000, abcam ab153710) to detect yeast eEF1A or an anti-human eEF1A2 antibody (1:1000, abcam 153714) to detect eEF1A2 for 1 h in TBST plus 1% milk. Secondary anti-rabbit-IgG conjugated to HRP was used to detect hybridisation (1:5000, Sigma) and was developed using enhanced chemiluminesence.
